# Practices in pelvic organ prolapse operations among surgeons: an international survey identifying needs for further research

**DOI:** 10.1007/s00192-016-2978-8

**Published:** 2016-02-19

**Authors:** Stephen O’Brien, Anudeep Dua, Monika Vij

**Affiliations:** Department of Obstetrics and Gynaecology, North Bristol NHS Trust, Southmead Hospital, Bristol, BS10 5NB UK; School of Clinical Sciences, University of Bristol, Bristol, UK; Department of Obstetrics & Gynaecology, Plymouth Hospitals NHS Trust, Derriford Hospital, Plymouth, PL6 8DH UK

**Keywords:** Prolapse, Technique, Training, Variation

## Abstract

**Objective and hypothesis:**

Our aim was to identify variation in surgical technique for treating pelvic floor disorders looking specifically at differences in approach between subspeciality trained urogynaecologists and general gynaecologists. We hypothesised that speciality trained surgeons would have a more uniform operative technique. We did not make a hypothesis about which operative areas would have the most variation overall.

**Methods:**

We performed a single-timepoint online survey of members of the International Urogynaecological Association (IUGA). Probability of difference from mean is presented as a raw value and significance of difference of means between surgical cohorts was calculated using the* t* test for independent variables.

**Results:**

We received 205 responses from 118 general gynaecologists and 87 from subspecialty trained urogynaecologists (8 % response rate) to 27 questions concerning operative steps in four common urogynaecological operations. Surgeons had low levels of variation. The probability of any surgeon providing a different answer from the mode of their cohort was not significant within or between surgeons with and without subspeciality training (*p* = 0.47). Two areas with high levels of variation between surgeons were identified (probability of variation >0.5). These were: “In order to reduce cystocele, do you plicate the fascia covering the bladder or use vaginal tissue?” and “Would you usually plicate the rectovaginal facial septum to the vault?”

**Conclusions:**

Most urogynaecological surgeries were of similar technique; however there were two areas of significant variation between surgeons that may affect outcomes and warrant further study.

## Introduction

Female pelvic floor disorders (PFD) involves both pelvic organ prolapse (POP) and urinary incontinence (UI). These disorders have a serious impact on patients’ quality of life (QoL). Several types of procedures with different surgical approaches have been described to correct PFD, but there is insufficient information to provide evidence-based recommendations regarding the optimal technique and materials [1], and trials are ongoing to identify optimal techniques. These trials are commonly designed to answer a debate regarding which procedure is most efficacious for a defined clinical problem, such as what type of sling should be used for treating stress urinary incontinence (SUI) [2]. However, variation exists concerning which are the most useful or common operations for managing POP. One variation is at the country level. For example, surgeons in Germany performed approximately four times as many POP procedures relative to incontinence procedures (such as bladder slings) than surgeons in France [3]. While this level of variation is well described, the literature does not address the question of where variation exists in surgical technique within defined operations. This is significant, as different techniques in the same operation could affect outcomes, with some surgeons believing a manoeuvre to be a routine step within a procedure and others disagreeing. Future research should take account of areas of intraoperative variation, but these are not clearly mapped in the literature, and this survey was designed to identify such areas to aid in future research regarding to either take account of these areas of variation or explicitly target them with randomised controlled trials to determine the magnitude of their effect on reported outcomes.

## Materials and methods

This was a single-timepoint online survey of surgical operators. All members of the International Association of Urogynaecologists (IUGA) were sent a single electronic mailing of the questionnaire containing closed-answer options for each question. Ethical review was not sought, as this was a survey of professionals and did not include individual patient information. The survey was designed in line with Cochrane recommendations and comprised 27 short questions on a white background pertaining to four common operations. Closed questions were used, as they are more likely to elicit responses [4]. . Operations examined were vaginal hysterectomy, anterior vaginal repair, posterior vaginal repair and transvaginal tape (TVT) use. Choice of questions was pragmatic, selected by a group of in-training and senior urogynaecologists, and covered domains in which there was felt to be variation in current practice. For example, surgeons were asked: “In a vaginal hysterectomy, how do you usually support the vault?” They were then asked to choose one of four answers: no support; uterosacral ligament-to-vault plication; broad ligament and uterosacral ligament-to-vault plication; round ligament, broad ligament and uterosacral ligament to vault plication. The survey also included questions about type and length of follow-up. All questions and responses are listed in Tables 1–5. Answers are presented using descriptive and subsequently relational statistics. Answers were analysed for probability of difference from the mean of the surgeon’s cohort, grouped by level of urogynaecologic training (subspecialty or not subspeciality trained). Difference between cohorts was analysed using the* t* test for independent variables.

**Table 1:** Responses in percentage terms from questions concerning posterior vaginal repairs

Questions	Answers	Subspecialist (87) (%)	Not subspeciality trained (118) (%)
Would you usually plicate the rectovaginal facial septum to the vault?	Always	37.3	28.7
	Sometimes	56.6	47.0
	Never	6.0	24.3
Do you usually perform a perineorrhaphy at the same time?	Always	44.6	49.6
	Sometimes	55.4	48.7
	Never	0.0	1.7
Do you plicate the levator ani together?	Always	12.0	14.8
	Sometimes	48.2	47.0
	Never	39.8	38.3
Which suture material would you usually use for fascial plication?	Vicryl 2.0	36.1	45.6
	PDS 2.0	26.5	22.8
	Monocryl 2.0	3.6	3.5
	Vicryl 1	8.4	16.7
	Vicryl 0	22.9	9.6
	Other	2.4	1.8

*PDS* polydioxanone

**Table 2:** Responses from questions concerning vaginal hysterectomy

Questions	Answers	Subspecialist (87) (%)	Not subspeciality trained (118) (%)
In a vaginal hysterectomy, how do you usually support the vault?	No support	1.2	2.7
	U-S ligaments to vault	67.9	77.9
	Broad and U-S ligament to vault	17.9	9.7
	Round, broad and U-S ligament to vault	13.1	9.7
In a vaginal hysterectomy, what suture method would you usually use to close the vault?	Interrupted sutures	36.8	28.7
	Continuous sutures, locked	36.8	50.4
	Continuous sutures, not locked	26.4	20.9
In a vaginal hysterectomy, which suture material would you usually use to close the vault?	Vicryl 2.0	31.4	35.3
	PDS 2.0	2.3	0.0
	Monocryl 2.0	4.7	2.6
	Vicryl 1	20.9	43.1
	Vicryl 0	38.4	16.4

*U-S* uterosacral

**Table 3:** Responses from questions concerning anterior repair

Questions	Answers	Subspecialist (87) (%)	Not subspeciality trained (118) (%)
Which form of fascial plication would you usually perform?	Continuous suturing	19.5	23.4
	Interrupted suturing	80.5	76.6
In order to reduce cystocele, do you plicate the fascia covering the bladder or use vaginal tissue?	Vaginal tissue	12.2	16.2
	Bladder fascia	40.2	51.4
	Both	47.6	32.4
What kind of suture material would you usually use for fascial plication?	Vicryl 2.0	43.9	51.4
	PDS 2.0	25.6	23.4
	Monocryl 2.0	2.4	1.8
	Vicryl 1	7.3	12.6
	Other	3.7	3.6

*PDS* polydioxanone

**Table 4:** Responses from questions concerning transvaginal tape (TVT)

Questions	Answers	Subspecialist (87) (%)	Not subspeciality trained (118) (%)
Do you usually do hydrodissection retropublically with a spinal or epidural needle?	Always	58.2	59.0
	Sometimes	13.9	17.0
	Never	27.8	24.0
Do you usually deflect the urethra when inserting the tape introducers?	Always	74.7	76.0
	Sometimes	6.3	9.0
	Never	19.0	15.0
How many times do you perform a check cystoscopy after inserting tape?	1	72.2	67.0
	2	24.1	26.8
	>2	3.8	6.2
What material do you usually use to close the vaginal incision?	Vicryl 2.0	72.2	69.7
	PDS 2.0	1.3	1.0
	Monocryl 2.0	5.1	7.1
	Vicryl 1	5.1	7.1
	Vicryl 0	3.8	7.1
	Other	12.7	8.1
When seeing a postop patient in clinic, would you usually perform a vaginal examination?	Always	89.9	78.8
	Sometimes	7.6	20.2
	Never	2.5	1.0

*Postop* postoperative, *PDS* polydioxanone

**Table 5:** Responses from questions concerning follow-up

Questions	Answers	Subspecialist (76) (%)	Not subspeciality trained (107) (%)
Having performed a prolapse repair operation, would you usually insert a vaginal pack or catheter?	Pack only	14.5	2.8
	Catheter only	80.3	10.3
	Pack and catheter	16.7	86.9
		Subspecialist (6)	Not subspeciality trained (4)
How long after inserting the vaginal pack would you usually remove it?	<12 h	0.0	0.0
	12–24 h	83.3	50.0
	25–36 h	0.0	50.0
	>36 h	16.7	0.0
		Subspecialist (11)	Not subspeciality trained (13)
What kind of catheter would you insert?	Transurethral	90.9	92.3
	Suprapubic	0.0	0.0
	Both, dependent on patient and surgery	9.1	7.7
		Subspecialist (12)	Not subspeciality trained (12)
How long after inserting the catheter would you usually remove it?	<12 h	41.7	50.0
	12–24 h	41.7	25.0
	25–36 h	16.7	16.7
	>36 h	0.0	8.3
		Subspecialist (80)	Not subspeciality trained (108)
Having performed a routine prolapse repair, how would you usually follow-up your patients?	Clinic follow-up	95	89.8
	Telephone follow-up	3.8	0.9
	No routine follow-up	1.3	9.3
		Subspecialist (77)	Not subspeciality trained (95)
When following up your patients in clinic, what is your routine follow-up interval?	4 weeks	35.1	26.3
	5–8 weeks	44.2	46.3
	9–12 weeks	13	14.7
	>12 weeks	7.8	12.6
		Subspecialist (3)	Not subspeciality trained (3)
When following up your patients via telephone, what is your routine postop follow-up interval?	4 weeks	0	0.0
	5–8 weeks	33.3	33.3
	9–12 weeks	0	33.3
	>12 weeks	66.7	33.3

## Results

There were 205 responders to all questions: 118 from general gynaecologists and 87 from subspecialty trained urogynaecologists. This represents a 7.6 % response rate from the ~2700 members of the IUGA. Responses were received from 44 countries. The five countries with the most respondents were UK (45), USA (27), Australia (15), Netherlands (11) and Canada (10). Respondents were asked how many of the four procedures were performed each month in their units. For vaginal hysterectomies, posterior and anterior vaginal repairs, the most common answer was six to ten per month. For retropubic TVT, the most common response was one to five per month. In posterior vaginal repairs, subspecialists were more likely to plicate the rectovaginal facial septum to the vault than were nonspecialists (6% “Never” response rate vs. 24.3 % “Never” response rate). Other questions showed no significant differences in response between surgeons. All questions and answers are shown in Table 1.

In vaginal hysterectomy, surgeons showed broad agreement across all questions. Greatest disparity was shown in the choice of suture for closing the vault: subspecialists were more likely to use Vicryl 0 over Vicryl 1.0 or 2.0 (38.4 %, 20.9 % and 31.4 %, respectively) compared with not subspeciality trained (16.4 %, 43.1 % and 35.3 %, respectively). All questions and responses are shown in Table 2.

In anterior vaginal repairs, surgeons demonstrated intercohort disagreement over how to reduce cystocele. Surgeons were more likely than not to choose a different mode of cystocele reduction that the mode of their cohort. This intercohort variance was present in both specialist and not subspeciality trained groups. Full questions and responses are shown in Table 3.

In responses to questions concerning TVT, surgeons showed broad agreement both within and between cohorts. The probability of surgeons choosing a different technique from the mode of their cohort was 0.3 and 0.33 for specialists and not subspeciality trained, respectively. Full questions and responses are shown in Table 4.

In questions concerning follow-up, some areas demonstrated significant differences within cohorts for both specialists and not subspeciality trained surgeons. These questions and results are shown in Table 5.

Overall, surgeons had low levels of variation in operative techniques. Subspecialty trained surgeons had a similar level of intracohort variation in operative techniques compared with their nonsubspecialty trained colleges. The probability of a subspecialty trained surgeon providing a different answer to the mode of their cohort for any question was 0.385; for general gynaecologists, it was 0.381. This variance was not significant (*p* = 0.47). The probability of a surgeon performing a different technique than the mode of their cohort for individual operations is given in the Table 6.

**Table 6:** Probability of a surgeon performing a different technique than the mode used by their cohort for individual operations

	Vaginal Hysterectomy	Posterior repair	Anterior repair	TVT	Follow-up
Subspecialty	0.46	0.5	0.42	0.3	0.22
General	0.42	0.52	0.39	0.33	0.24

*TVT* transvaginal tape

There were two areas with high levels of variation between surgeons, regardless of level of training (probability of variation from modal answer >0.5). These were: “When performing an anterior repair, in order to reduce cystocele, do you plicate the fascia covering the bladder or use vaginal tissue?” and, when performing a posterior vaginal wall repair: “Would you usually plicate the rectovaginal facial septum to the vault?”

## Discussion

Our study demonstrated a high degree of agreement between surgeons in areas previously thought to have varied practice. We have shown that speciality trained urogynaecologists and general gynaecologists are not more or less variable in their intraoperative practice than each other, refuting our hypothesis. We have, however, highlighted two specific areas where there remains wide variation between surgeons: whether or not to plicate the rectovaginal septum to the vault during a posterior repair; and whether during an anterior repair the surgeon should, in order to reduce cystocele, plicate the fascia covering the bladder or use vaginal tissue. A randomized trial to assess the outcomes of adopting each of the management strategies in the cases above could therefore demonstrate potential patient benefit of one technique over another.

A literature search shows that this study is the only attempt to determine where variation exists within urogynaecological operations and to quantify its extent. As operations to treat POP increase in both absolute and relative frequency [5], so the importance of establishing how these operations are conducted in the real world increases. Previous studies within urogynaecology have assessed differences in outcomes following various different operative procedures; for example, there have been studies of differences in operative materials, such as bladder-neck slings [2], suture types and techniques for obstetric anal sphincter injury [6] and suture techniques in vaginal vault prolapse surgery [7]. These studies have generated useful findings, but their rationale for investigating the individuals concerned is pragmatic and limited by the interests of the authors according to what is generally perceived to be common practice. There has not yet been a prospective, systematic attempt to identify areas of variation with urogynaecology operations. Our study attempts to address this issue.

The strength of our study is that this is the largest study of surgeon-reported variation within urogynaecological operations conducted thus far, to the best of our knowledge. It was single timepoint in design and accessed the largest current self-identified population of surgeons performing urogynaecological operations. 

The study does, however, have several limitations inherent in its design. It is an online survey of members of a specialist society, and therefore subject to two levels of selection bias. It is unlikely to be representative of clinical practice of surgeons without a special interest in urogynaecology, and its findings should be interpreted with caution before being applied to all generally trained gynaecologists with a special interest in urogynaecology. Measures to negate the influence of nonresponse bias were considered by the authors, in line with recommendations around interpreting online survey responses [8]; however, performing either a sensitivity analysis of nonrespondents or conducting a multiple-imputation analysis were felt to be impractical and would not add value to the findings of the survey. No attempt was made to validate the questionnaire. This was for two reasons: Firstly, there is no gold standard of question to concept reliability in this area with which to compare our questions. Secondly, generating an adequate sample (20 participants) with which to test the questionnaire outside of the main studied sample was not possible given our resources. Surgeons were defined as subspecialty trained if they had completed formal urogynaecology subspecialty training. We acknowledge that this means that a number of senior surgeons who were trained before subspecialty training existed but are urogynaecological trainers and leaders in the field would have been included in the not subspeciality trained cohort. However, defining such surgeons is necessarily subjective, while whether or not respondents had completed subspecialty training is an objective characteristic. We therefore chose this as our differentiating point between cohorts. The response rate was low, at 7.4 %. This is, however, similar to other single-timepoint online surveys, and the absolute number of respondents is similar to other surveys in this area [9]. We recognise that, as there are many nonsurgeon members of the IUGA, our response rate from surgeon members of the IUGA is likely to be higher than 7.4 %, but as the IUGA is not able to provide a breakdown of its membership, we are unable to confirm or quantify this. Two areas (insertion of vaginal pack and/or catheter following surgery, and use of telephone follow-up) were stratified by previous answer and therefore have low numbers of respondents. This severely limits the validity of these results. Nearly one quarter of all respondents (45) were from a single country (the UK), and nearly half (100) were from primarily English-speaking countries. While this will reduce the applicability of results to all non-English-speaking countries, it does not invalidate the overall objective of the study, which was to gain a pragmatic overview of current practice. The survey did not gather data on respondents by age, years of training or individual numbers of procedures performed. While we recognise that some readers will feel this information would be important, we felt that the additional time it would take respondents would reduce the response rate; instead, data already gathered by respondents self-identifying their role and giving the number of procedures performed within their unit would fulfill this need. Some respondents may have been more familiar with the term “vaginal muscularis” rather than “fascia,” referring to the pubicervical or rectosigmoid fascia. However, we do not feel that surgeons who use this term routinely would be unfamiliar with the synonymous use of the term fascia.

We recognise that this is a study with limited generalisability, but we believe that it is sufficient for its stated purpose, which was to explore and outline general areas of variation within defined operations to guide future targeted research.

## Conclusions

Rates of surgical procedures to treat POP are increasing [5]. More trials are taking place comparing defined operations against each other (and against nonoperative strategies) to determine the most efficacious treatment. This survey shows that there exist some areas of variation within established operations, which could affect patient outcomes and should be recognised in any future trials. They may also be studied individually to determine the size of effect that the variation has on patient outcomes.
